# Diversity and Function of Microbial Community in Chinese Strong-Flavor Baijiu Ecosystem: A Review

**DOI:** 10.3389/fmicb.2018.00671

**Published:** 2018-04-09

**Authors:** Wei Zou, Changqing Zhao, Huibo Luo

**Affiliations:** College of Bioengineering, Sichuan University of Science and Engineering, Zigong, China

**Keywords:** strong flavor baijiu, microbial community, pit mud, ethyl hexanoate, systems biology

## Abstract

Strong flavor baijiu (SFB), also called Luzhou-flavor liquor, is the most popular Chinese baijiu. It is manufactured via solid fermentation, with daqu as the starter. Microbial diversity of the SFB ecosystem and the synergistic effects of the enzymes and compounds produced by them are responsible for the special flavor and mouthfeel of SFB. The present review covers research studies focused on microbial community analysis of the SFB ecosystem, including the culturable microorganisms, their metabolic functions, microbial community diversity and their interactions. The review specifically emphasizes on the most recently conducted culture-independent analysis of SFB microbial community diversity. Furthermore, the possible application of systems biology approaches for elucidating the molecular mechanisms of SFB production were also reviewed and prospected.

## Introduction

**FIGURE 1:**
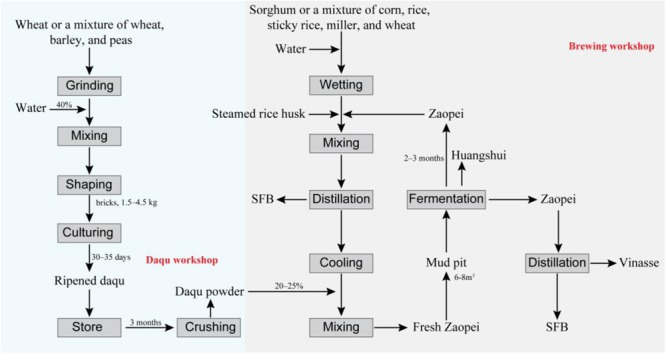
Workflow of the strong flavor baijiu (SFB) production. The technology used in SFB production is called “Back-slopping technique” (Zheng and Han, 2016). The raw material for SFB production are cereals, mostly sorghum or a mixture of corn, rice, millet, sticky rice, and wheat (Zheng and Han, 2016). Daqu is the saccharifying and fermentative agent used in the SFB production (Zheng et al., 2011). The production of daqu is a solid fermentation process with a natural inoculation of microorganisms originated from production environment and cereals materials, mainly involving three stages: material shaping, incubating, and drying (Zheng et al., 2011; Wu et al., 2014). The zaopei (fermented cereals) is mixed with sorghum, rice or other cereals and steamed rice husk and distilled for the SFB, then after cooling, the distilled solid residue was mixed with daqu powder, and fresh zaopei was obtained. The fresh zaopei was put into a mud pit (volume of 6–8 m^3^) and the top is covered with yellow mud so as to achieve and maintain an anaerobic environment (Jin et al., 2017). In mud pit, the saccharification and fermentation process occur simultaneously and last for 2–3 months (Xu et al., 2010; Zheng and Han, 2016). Then, the fermented zaopei is took out. The zaopei in the upper layer (also name *mianzao*) is distilled to obtain SFB. The distilled solid residue is vinasse. The other zaopei (also name *niangzao*) is mixed with cereals and enters into another cycle for SFB production. Huangshui (HS) is the brown viscous liquid which is formed by the liquid that permeates to the bottom of mud pit during the fermentation process (Feng et al., 2017).

Strong-flavor baijiu (SFB), also called Luzhou-flavor liquor, is the most popular Chinese baijiu that is known to exist since the past many centuries (Zheng and Han, 2016; Jin et al., 2017; Xu et al., 2017). Latest data reveal that the total yield of SFB has reached 9.1 million tons per annum (Wang, 2016). SFB is usually produced with the help of a typical method of natural solid fermentation that uses daqu as the main saccharification agent (**Figure 1**). The materials of SFB are cereals, mostly sorghum or a mixture of corn, rice, millet, sticky rice, and wheat. The fermentation process is anaerobic, carried out in a mud pit (normally with a volume of 6–8 m^3^) and lasts for 60–90 days (Zheng and Han, 2016). SFB is known to contain over 1300 different kinds of flavoring compounds (Yao et al., 2015). It has a characteristic fragrant flavor, soft mouthfeel, and long-lasting aftertaste (Zheng and Han, 2016). Like beer and wine, the composition of the flavoring compounds in SFB is determined by its microbial diversity (Bokulich et al., 2012). However, the open fermentation environment and complex microbial composition of the procedure makes it difficult to elucidate the exact specifications of SFB production.

At present, the SFB industry faces many constraints, such as long term fermentation time, unstable quality, lack of knowledge of the exact mechanism of formation of the flavoring compounds, low mechanization, and labor-intensive procedure (Jin et al., 2017). The key factors that can help in solving these problems can only be determined by procuring comprehensive understanding of the microbial community composition of the SFB ecosystem. Furthermore, it is crucial to understand the functional dynamics of the dominant microbial strains present in SFB ecosystem. In an effort to achieve the same many microbes have already been isolated and cultured and their physiological and metabolic functions studied. The information thus obtained provided some crucial insights into the chemical nature and mechanism of formation of SFB flavoring compounds. Recent introduction of application of culture-independent methods, such as polymerase chain reaction denaturing gradient gel electrophoresis (PCR-DGGE) and Illumina sequencing have definitely enhanced our knowledge regarding the diversity and structure of the microbial community of SFB ecosystem (Ding et al., 2014b; Sun et al., 2016; Huang et al., 2017a). In this review paper, recent findings pertaining to the diversity and function of microbial community of SFB ecosystem are discussed. The article most specifically emphasizes on the results discovered from culture-independent method based research studies.

## Microbial Diversity of Cultured Microorganism

Isolation and culture of microorganisms from the SFB ecosystem was initially started in the 1960s (Wu et al., 1991). Since then, many microbial strains have been screened and identified (**Table 1**). As far as bacterial diversity is concerned, two elaborate systematic studies encompassing the isolation and culture of bacteria from the SFB ecosystem (including, workshop environment, daqu, pit mud, and zaopei) were performed (Zhou et al., 2010; Wang T. et al., 2011). A total of 34 genera of bacteria were identified, of which *Bacillus, Streptomyces, Lysinibacillus, Staphylococcus, Rummeliibacillus, Brevibacillus*, and *Brachybacterium* were the most dominant (Zhou et al., 2010; Wang T. et al., 2011). Other dominant genera included *Weissella, Pediococcus* (Yang J.-G. et al., 2017), *Lactobacillus, Acetobacter* (Ming et al., 2013) from daqu; and *Sporolactobacillus, Clostridium, Mycobacterium*, and *Flavobacterium* from pit mud (Yue et al., 2007). In addition, six new species of bacteria [*Paenibacillus vini* (Chen et al., 2015), *Bacillus vini* (Ma et al., 2016), *Lysobacter zhanggongensis* (Zhang et al., 2017), *Clostridium swellfunianum* (Liu et al., 2014), *C. luticellarii* (Wang et al., 2015), and *C. liquoris* (Yin et al., 2016)] were detected and identified from the SFB ecosystem.

**Table 1:** Microorganisms isolated and identified from strong flavor baijiu ecosystem through culture-dependent methods.

Samples	Places	Isolated species/genus	Reference
Mature daqu	Gansu	*Bacillus licheniformis, Bacillus cereus, Bacillus subtilis, Bacillus sonorensis, Brevibacillus* sp., *Bacillus amyloliquefaciens, Bacillus atrophaeus*	Lin et al., 2012
Mature daqu	Luzhou, Sichuan	*Aspergillus, Monascus, Rhizomucor, Lichtheimia, Penicillium, Paecilomyces, Saccharomyces cerevisiae, Debaryomyces, Wickerhamomyces anomala, Sporidiobolus pararoseus, Merimbla ingelheimensis, Talaromyces, Cercospora, Cladosporium, Acremonium impicatum, Neurospora*	Luo et al., 2013
Mature daqu	Luzhou, Sichuan	Dominant bacteria: *Lactobacillus, Acetobacter, Bacillus, Brettanomyces*. Dominant fungi: *Candida, Dekkera, Mucor, Aspergillus, Rizopus*	Ming et al., 2013
Mature daqu	Hunan	*Bacillus licheniformis, Bacillus subtilis Bacillus amyloliquefaciens*, and *Bacillus cereus* were dominant bacteria those possessed high activities of a-amylase and glucoamylase	Li et al., 2014
Mature daqu	Luzhou, Sichuan	Ester-producing yeasts: *Candida* sp., *Hansenula* sp., *Brettanomyces* sp., *Dekkera* sp.	Xu, 2016
Daqu fermented for 5, 7, 10, 25, and 90 days	Luzhou, Sichuan	The dominant bacteria, yeast, and mold strains in mature daqu were *Bacillus subtilis* subsp. *inaquosorum, Saccharomycopsis fibuligera*, and *Lichtheimia ramosa. Mucor circinelloides* f. *circinelloides* was strongly correlated with protease, and saccharifying enzyme activity was mainly correlated with *Rhizopus oryzae*	Yang J.-G. et al., 2017
Pit mud	Luzhou, Sichuan	Facultative anaerobes: *Bacillus, Sporolactobacillus*, Pse*ud*omonas, *Clostridium, Mycobacterium, Pseudomonas, Microbacterium, Corynebacterium, Flavobacterium*	Yue et al., 2007
Pit mud	He Bei, Bei Jing, Si Chuan	*Pediococcus pentosaceus, P*. *acidilactici, P. stilesii, P. dextrinicus, P. parvulus, P. inopinatus, P. ethanolidurans, P. damnosu*s, *P. cellicola*	Liu et al., 2007
Pit mud	–^#^	*Schizosaccharomyces pombe, Kluyveromyces thermotolerans, Hansenula polymorpha, Saccharomyces cerevisiae, Zygosaccharomyces rouxil*	Yang et al., 2011
Pit mud	–	Caproic acid producing strains: *B. megaterium, B. fusiformis, B. licheniformis*	Zhao H. et al., 2012
Pit mud from 1 and 10 years old pits	Sichuan	*Bacillus, Rummeliibacillus, Clostridium, Paenibacillus*	Wang C.-D. et al., 2014
Pit mud	Luzhou, Sichuan, Suqian, Jiangsu, Yibin, Sichuan	*C. celerecrescens, C. indolis, C. tyrobutyricum, C. amylolyticum, C. butyricum, C. sartagoforme*, and *C. kluyveri*	Hu et al., 2015
Pit mud from 45 and 65 years old pits	Sangqiu, Henan	*C. celerecrescens, C. cochlearium, C. carboxidivorans, C. sporogenes, C. sartagoforme, C. thermopalmarium, C. aurantibutyricum, C. butyricum*	He et al., 2017
Pit mud from 100 years old pit	Luzhou, Sichuan	*Lysinibacillus sphaerieus, Brevibacillus brevis, Paenibacillus larvae* subsup. *pulvifacies*	Liu Y. et al., 2017
Zaopei fermented for 1, 4, 7, 14, 24, 34, 44 days	–	*Corynebacterium xerosis, Staphylococcus auricularis, Bacillus subtilis, Bacillus megaterium, Bacillus cereus group*, and *Paenibacillus macerans* produced lactic acid	Yao et al., 2010
Zaopei	Suqian, Jiangsu	*Lactococcus garvieae, Bacillus amyloliquefaciens, Pediococcus acidilactici, Staphylococcus pasteuri* produced citrulline from arginine in high efficacy	Qiu et al., 2016
Zaopei fermented for 0, 4, 8, 12, 20, 30, 44 days	Luzhou, Sichuan	*Acetobacter malorum, Acetobacter cerevisiae, Bacillus methylotrophicus, Acetobacter aceti, Acetobacter estunensis, Acetobacter pasteurianus* subsp. *pasteurianus, Bacillus vanillea* were the main species in initial fermentation stage, *Bacillus amyloliquefaciens* subsp. *plantarum, Bacillus methylotrophicus, Bacillus atrophaeus, Bacillus subtilis* subsp. *inaquosorum, Lactobacillus buchneri, Lactobacillus paracasei* subsp. *tolerans, Bacillus vanilla* were the dominant species in medium fermentation stage. *Bacillus amyloliquefaciens* subsp. *plantarum, Bacillus subtilis* subsp. *inaquosorum, Bacillus sonorensis, Bacillus methylotrophicus, Bacillus vanillea, Bacillus atrophaeus, Gluconobacter cerinus* were dominant in late fermentation stage	Dou et al., 2017
Zaopei fermented for 0, 4, 8, 12, 20, 30, 44 days	Luzhou, Sichuan	*Candida rugopelliculosa, Pichia fermentans, Naumovozyma castellii, Torulaspora delbrueckii, Saccharomyces cerevisiae, Pichia membranifaciens* were dominant in 0, 4 days*; Saccharomyces cerevisiae, Candida humilis, and Kazachstania exigua* were dominant in 8 days; *Naumovozyma castellii* and *Saccharomyces cerevisiae* were dominant in 12 days; *Saccharomyces cerevisiae* were dominant in 20, 30 days*; Candida ethanolica* was found in 44 days	Yang J. et al., 2017
SFB ecosystem^∗^	Yibin, Sichuan	*Bacillus, Lysinibacillus, Staphylococcus, Rummeliibacillus, Brevibacillus, Brachybacterium*	Zhou et al., 2010
SFB ecosystem	Yibin Sichuan	*Streptomyces, Massilia, Nocardiopsis*	Zhang et al., 2011
SFB ecosystem	Yibin, Sichuan	Dominate genera: *Bacillus, Streptomyces, Lysinibacillus, Staphylococcus*	Wang T. et al., 2011
SFB ecosystem	Yibin, Sichuan	*Streptomyces utabilis, S. vinaceusdrappus, S. coelicoflavus, S. violascens* produced ethyl lactate, caproic acid as the main volatile products	You and Wang, 2012
SFB ecosystem	Yibin, Sichuan	*Bacillus methylotrophicus, B. cereus, B. megaterium, B. aryabhattai, B. thuringiensis, B. tequilensis, Rhodococcus ruber, Brevudimonas naejangsanensis, Pseudomonas koreensis, Lysinibacillus sphaericus* produced soy-like aroma	You et al., 2014

*^#^, not available. ^∗^SFB ecosystem includes Daqu and fermentation workshop air, daqu, pit mud, zaopei.*

On the other hand, *Wickerhamomyces, Saccharomycopsis, Meyerozyma, Candida, Pichia, Cryptococcus, Brettanomyces, Dekkera, Issatchenkia, Debaryomyces, Saccharomyces, Rhodotorula, Schizosaccharomyces, Kluyveromyces, Hansenula*, and *Zygosaccharomyces* were the yeast genera that were isolated and identified from the SFB ecosystem (Wu et al., 2006; Ming et al., 2013; Zhang X. et al., 2015; Yang J.-G. et al., 2017), among which *Saccharomycopsis* was the most dominant in daqu (Yang J.-G. et al., 2017); and *Issatchenkia, Pichia*, and *Candida* in zaopei (Wu et al., 2006; Zhang X. et al., 2015). *Lichtheimia, Aspergillus, Penicillium, Rhizomucor, Mucor, Rhizopus, Monascus, Emericella, Cladosporium*, and *Gibberella* were the mold genera isolated and identified from the ecosystem (Pu et al., 2012; Wang et al., 2012; Ming et al., 2013; Zhang X. et al., 2015; Yang J.-G. et al., 2017). It was also observed that among these mold genera, *Penicillium* and *Aspergillus* were the most dominant in the brewing workshop (Wang et al., 2012), while *Lichtheimia* was dominant in daqu (Yang J.-G. et al., 2017). Similarly, *Aspergillus, Monascus*, and *Rhizopus* were found to be most dominant in zaopei (Zhang X. et al., 2015). In addition, three archaea were also isolated. Two of them were identified as *Methanobacterium bryantii* (Wu et al., 1990) and *Methanobacterium formicicum* (Wang et al., 2010).

## Physiological Traits of Cultured Microorganisms

The production of SFB involves three basic functional processes: saccharification, alcohol fermentation, and flavor compound formation. Saccharification is achieved through extracellular enzymes (amylase, glucoamylase, protease, cellulose, and lipase) that are secreted by the microorganisms present in the SFB ecosystem, mostly in daqu. For amylase, *Bacillus* and *Staphylococcus* are the main bacterial producers, and for glucoamylase, *Bacillus, Enterobacter*, and *Weissella* are the main bacterial producers (Lin et al., 2012; Li et al., 2014; Yang J.-G. et al., 2017). In addition, *Rhizopus*, and *Lichtheimia* were found to produce amylase; and *Aspergillus, Mucor, Penicillium, Rhizopus, Gibberella*, and *Cladosporium*, produced glucoamylase (Pu et al., 2012; Li et al., 2014; Yang J.-G. et al., 2017). Some of the chief microbial cellulase producers included, *Bacillus, Penicillium, Aspergillus*, and *Alternaria* (Zeng et al., 2016). In addition, *Staphylococcus gallinarum, Mucor circinelloides* f. *circinelloides* were found to secrete protease (Yang J.-G. et al., 2017); and *Rhizopus chinensis* lipase (Wang D. et al., 2013).

Yeasts are the main ethanol producers in SFB ecosystem. *Saccharomyces cerevisiae* was the main ethanol producer (Hu et al., 2017) and was dominant in the zaopei fermentation process (Yang J. et al., 2017). Ethyl hexanoate was identified as the most typical flavoring compound of SFB (Zheng and Han, 2016). In an effort to elucidate the process of its formation many hexanoate producing microorganisms have been identified and studied. Generally, hexanoate is produced by the members of the genus *Clostridium* by using glucose, lactate, ethanol and acetic acid as substrates (De Araújo Cavalcante et al., 2017). Some of the most common hexanoate producing *Clostridium* species include, *C. kluyveri* (Hu et al., 2015), *C. lushun* (Wu and Yi, 1986), *Clostridium* Sp. W1 (Xue et al., 1988), and *C. celerecrescens* (Zhao H. et al., 2012; Xue and Xue, 2016). In addition, *Bacillus megaterium, Bacillus fusiformis*, and *Bacillus licheniformis* are also known to produce hexanoate (Zhao H. et al., 2012). Ethyl lactate, ethyl acetate, and ethyl butanoate are another representative compounds in SFB. Lactic acid bacteria (LAB) were found as the dominant microorganisms in mature daqu (Ming et al., 2013) and zaopei (Dou et al., 2017), contributing to the lactic acid production. Other lactic acid producers in SFB ecosystem included: *Corynebacterium xerosis, Staphylococcus auricularis, Bacillus subtilis, Bacillus megaterium, Bacillus cereus group*, and *Paenibacillus macerans* (Yao et al., 2010). Acetic acid was produced via genus *Acetobacter*, which was found as one of the main genera in the initial stage of zaopei fermentation (Dou et al., 2017). Furthermore, specific members of the genus *Clostridium* were also identified to biosynthesize acetate, butanoate, and lactate from various substrates, such as sugar, starch, and cellulose (Dürre, 2016). In addition, *Bacillus, Lysinibacillus, Sporosarcina, Staphylococcus* isolated from SFB ecosystem were found to produce different types of organic acids (Tang et al., 2013). These organic acids, together with ethanol get esterified into corresponding ethyl esters via a reaction catalyzed by esterases. Further investigations revealed that species of *Penicillium, Aspergillus, Emericella, Rhizopus, Cladosporium, Mucor, Hansenula, Candida, Hansenula, Brettanomyces*, and *Dekkera* isolated from SFB ecosystem are capable of secreting esterases (Wang et al., 2012; Xu, 2016). Other flavoring compounds of SFB, such as ketones, pyrazine, and phenylethanol were also found to be produced by *Bacillus, Wickerhamomyces anomalus* (Ming et al., 2015; Zhou et al., 2016).

In addition to the beneficial microorganisms that were responsible for the characteristic taste and flavor of SFB production, certain other unpleasant flavoring compound producing strains were also detected. *p*-Cresol was identified as the major off-odor and toxic component present in SFB. It was later found to be produced by *C. butyricum, C. tyrobutyricum, C. aminovalericum, C. ultunense*, and *C. purinilyticum* (Du et al., 2017; Liu B. et al., 2017). Ethyl carbamate is another potential carcinogenic compound that was found to be present in SFB. Citrulline, one of the precursors of ethyl carbamate (Wang H. et al., 2014) that was found to be aacumulated (by using argine) by *Lactococcus garvieae, Bacillus amyloliquefaciens, Pediococcus acidilactici*, and *Staphylococcus pasteuri* (Qiu et al., 2016). On the other hand, geosmin that has an earthy off-flavor was found to be produced by *Streptomyces* isolated from SFB ecosystem (Wang T. et al., 2011; Du and Xu, 2012).

## Microbial Diversity Revealed by Culture-Independent Methods

Although the research studies based on traditional microbial culture methods helped in gaining preliminary insights into the microbial diversity of SFB ecosystem, it was realized that these procedures were unable to study a large number of microbes whose isolation and culturing is difficult to achieve with the help of adept microbiological methods (Kaeberlein et al., 2002). This led to the implementation of various culture-independent methods that were directed toward attaining comprehensive understanding of microbial diversity of the SFB ecosystem (**Table 2**). PCR-DGGE and sequencing technology were mostly used to investigate the microbial diversity of the SFB ecosystem via the culture-independent approach.

**Table 2:** Studies on microbial diversity of strong flavor baijiu ecosystem with culture-independent methods.

Samples	Locations	Methods	Main species or results	Reference
Mature daqu	Haozhou, Anhui	PCR-cloning	*Lactobacillus, Pantoea, Enterobacter, Klebsiella, Leuconostoc, Erwinias, Pseudomonas, Bacillus licheniformis*	Bin et al., 2011
Mature daqu	Huaian, Jiangsu; Mianzhu, Sichuan	PCR-DGGE	Dominant bacteria: lactic acid bacteria and *Staphylococcus xylosus*. Dominant yeasts *Saccharomycopsis fibuligera* and *Pichia anomala*. Dominant molds: *Rhizomucor miehei, Absidia blakesleeana* and *Aspergillus terreus*	Wang H.Y. et al., 2011
Mature daqu	Luzhou, Sichuan	Nested PCR-DGGE	Dominant bacteria: Lactic acid bacteria and *Bacillus* were. Dominant yeasts: *Saccharomycopsis fibuligera, Wallemia sebi, Wallemia muriae*, and *Pichia subpelliculosa.* Dominant molds: *Aspergillus*	Zhang et al., 2014
Mature daqu	Sichuan	Cloning	Main bacteria: *Thermoactinomyces sanguinis, Enterobacter cloacae, Pantoea agglomerans*, and uncultured bacteria. Main molds: *Aspergillus glaucus, Thermomyces lanuginosus, and Thermoascus crustaceus*	Gou et al., 2015
Mature daqu	40.02°N; 28.88° N; 28.55°N	Gene clone libraries	*Staphylococcus gallinarum, Staphylococcus saprophyticus* were only found in southern daqu. *Saccharomycopsis fibuligera* and *Lichtheimia ramosa* were dominated fungi; *Bacillus licheniformis, S. fibuligera* and one uncultured bacterium were detected in all samples	Zheng X.W. et al., 2015
Daqu fermented 0, 2, 4, 6, 8, 10, 12, 17, 27, and 32 days	Luzhou, Sichuan	454 pyrosequencing and Illumina MiSeq sequencing	In the first 4 days of fermentation, most bacterial taxa, and several fungal taxa containing *Candida, Wickerhamomyces*, and unclassified *Dipodascaceae*, and *Saccharomycetales*, grow well. From day 4 to day 12, thermotolerant taxa including *Bacillus*, unclassified *Streptophyta, Weissella, Thermoactinomyces, Thermoascus*, and *Thermomyces* survived or kept on growing. Lactic acid bacteria related to *Weissella, Leuconostoc*, and *Lactobacillus* were dominant bacteria through fermentation, while *Bacillus* became a dominant genus after 10 days of fermentation. *Thermoascus, Candida, Wickerhamomyces*, and *Thermomyces* were dominant fungal genera through fermentation	Xiao et al., 2017
Daqu fermented 0, 3, and 9 days, and mature daqu	Yibin, Sichuan	454 pyrosequencing	*Lactobacillales* became dominant during the first 3 days and then decreased markedly. *Bacillales* became dominant in 9 days and mature daqu. *Staphylococcus* spp. and *Chryseobacterium* spp. were the most abundant genera in common across the 4 samples. *Acetobacter* and *Lactobacillus* increased quickly from 0 day to 3 days and decreased later. *Saccharomycetales* were predominant fungi after 3 days of incubation. *Saccharomycetales* and no-rank *Eukaryota* were dominant in 9 days. *Eurotiales* became the dominant in mature daqu. *Pichia* was the dominant genus.	Huang et al., 2017b
Pit mud from 20, 100 and over 300 years old pits	Luzhou, Sichuan	PLFA	The microbial community of pit mud was composed of bacteria, actinomycetes and fungi, with Gram-positive bacteria and anaerobic bacteria being dominant. As the pit age increased, pit mud biomass increased and the microbial community shifted to Gram positive bacteria	Zhao et al., 2012
Pit mud from 20, 50, 100, 200, and 300 years old pits	Luzhou, Sichuan	PCR-DGGE and PLFA	Dominant bacteria: Clostridiales, Lactobacillales, and Bacillales; Dominant yeasts: *Wickerhamomyces, Kluyveromyces, Pichia*, and *Pichia anomala*	Zheng et al., 2013
Pit mud from 1, 2, 3, and 4 years old pits	Luzhou, Sichuan	PCR-DGGE and FISH	Dominant bacteria: Leuconostocaceae, Clostridiaceae, Lactobacillaceae, Moraxellaceae, Enterococcaceae, Lachnospiraceae, Comamonadaceae, Sphingomonadaceae, and Ruminococcaceae; Dominant archaea: *Methanobrevibacter, Methanobacterium*, and *Methanoculleus*	Ding et al., 2014a
Pit mud from the wall and bottom of 200 years old pits	Luzhou, Sichuan	PCR-DGGE	*Clostridium* was the dominant eubacteria; *Methanoculleus*, and *Methanosaeta* were the main archaea; eubacteria and archaea community diversities in samples from the bottom were almost higher than that from the wall; Acinetobacter was found in all samples from the wall, but not the bottom	Ding et al., 2014b
Pit mud from 50, 140, 220, 440 years old pits	Luzhou, Sichuan	Metagenomics sequencing	The microbial communities in all the pits were dominated by *Firmicutes*. The youngest pit had the highest proportions of *Gammaproteobacteria* and opisthokonts. The abundances of *Euryarchaeota* and *Bacteroidetes* increased as the age of pit mud increasing	Guo et al., 2014
Pit mud from the bottom of aged and aging pits	Anhui	16S rRNA gene clone libraries and quantitative real time PCR	*Firmicutes* and *Chloroflexi* predominated in the aged pit mud while *Firmicutes* and *Bacteroidetes* predominated in the aging pit mud. *Chloroflexi* and *Actinobacteria* were only detected in the aged pit mud. The quantity of *Actinobacteria* in the aged pit mud was 29 times as much as in the aging pit mud	Luo et al., 2014a
Pit mud from aged and aging pits	Anhui	Gene clone libraries and amplified ribosomal DNA restriction analysis	*Bacteroidetes* and *Firmicutes* predominated in both the aged and aging pit mud, but *Synergistetes* and *Actinobacteria* were only detected in the aged pit mud. The *Methanosaeta* dominated in the aged pit mud, while the *Methanosarcina* predominated in the aging pit mud	Luo et al., 2014b
Pit mud from 1, 10, 25, and 50 years old pits	Mianzhu, Sichuan	Pyrosequencing	Dominant genera include *Petrimonas*, unclassified *Clostridiaceae, Methanoculleus, Methanosarcina, Methanobacterium, Methanobrevibacter, Lactobacillus, Clostridium* IV, *Sedimentibacter, Syntrophomonas, Spirochaetes* SHA-4, *Methanobrevibacter*, and unclassified *Porphyromonadaceae, Anaerobrancaceae*, and *Ruminococcaceae*	Tao et al., 2014
Zaopei, pit mud, and huangshui from 2, 10, and 30 years old pits	Yibin, Sichuan	PCR-DGGE and PLFA	All the eubacteria belonged to *Lactobacillaceae, Clostridiaceae, Porphyromonadaceae, Synergistaceae*, and *Acetobacteraceae*. *Lactobacillaceae* was dominant eubacteria in the ZP, while *Clostridiaceae* was dominant eubacteria in the PM and HS, respectively. *Methanosaeta, Methanocorpusculum, Methanobrevibacter, Methanobacterium*, and *Methanoculleus* were the majority of archaea. *Methanosaeta*, increased gradually in the PM and HS with pit age, and decreased in the ZP. *Pichia* was dominated in fungal community	Ding et al., 2015
Pit mud from aged and aging pits	Sichuan	PCR-DGGE and quantitative PCR	*Clostridiales* was dominant in aged pit mud while *Bacillales* and *Lactobacillales* were dominant in aging pit mud	Liang et al., 2015
Pit mud from 1, 50, 100, and 300 years old pits	Luzhou, Sichuan	PCR-DGGE and FISH	*Methanobacteriales* dominated in low-age (1 and 50 years) pit mud. *Methanomicrobiales* dominated in old age (100 and 300 years) pit mud	Wu et al., 2015
Pit mud from 1 and 2 years old pits	Yibin, Sichuan	nested PCR-DGGE, PLFA, PLEL, FISH	Dominated bacteria: *Clostridiales, Lactobacillales, Bacteroidales*, and *Rhizobiales*. Dominated archaea: *Methanomicrobiales* and *Methanosarcinales*. Dominated fungi: *Saccharomycetales* and *Eurotiales*	Zhang L. et al., 2015
Pit mud from 30 and 300 years old pits	Luzhou, Sichuan	iTRAQ-based proteomic approach and high-throughput sequencing	The aroma-forming functional proteins in 300-year pit mud were highly expressed with much higher content than that of 30-year pit mud, *Firmicutes* and *Methanobacterium*, were important components of aroma-forming functional colonies in the pit muds	Zheng et al., 2015
Degraded, normal, and high quality pit mud	Jiangsu	Illumina MiSeq sequencing	Core genera in all samples included: *Lactobacillus, Ruminococcus, Caloramator, Clostridium, Sedimentibacter, Syntrophomonas, Sporanaerobacter, Pelotomaculum*, T78, *Prevotella*, Blvii28 group, *Methanobacterium, Methanobrevibacter, Methanosaeta, Methanoculleus, Methanosarcina*, and *Nitrososphaera*. Clostridia, Bacteroidia, Methanobacteria, and Methanomicrobia, may play important roles in pit mud ecosystem stability, which may be destroyed with rapidly increased levels of lactic acid bacteria (*Lactobacillus, Pediococcus*, and *Streptococcus*)	Hu et al., 2016
Matured and degenerated pit mud	Sichuan, Anhui	PCR-DGGE and qPCR	Bacterial community in the degenerated pit mud did not change with different regions. Bacterial community in the matured pit mud from different regions could be different. *Actinobacteria* could serve as an indicator to distinguish pit muds	Liang et al., 2016
Pit mud from 5 and 100 years old pits	Luzhou, Sichuan	PCR-DGGE, illumina MiSeq sequencing	*Rhizopus, Aspergillus, Phoma, Trichosporon, Candida, Thermoascus, Wickerhamomyces, Penicillium, Thermomyces, Debaryomyces, Saccharomyces, Malassezia, Mucor, Davidiella, Wallemia, Toxicocladosporium, Fusarium, Pichia*, and *Cladosporium* were identified as core genera. *Rhizopus, Phoma*, and *Trichosporon* were relatively richer in the 5-year PM samples, and *Aspergillus* and *Candida* were rich in the 100-year PM samples	Liu M. et al., 2017
Pit mud from 30 years old pits	Mianzhu, Sichuan	Illumina sequencing	The dominant prokaryotic phyla were *Firmicutes, Euryarchaeota, Bacteroidetes, Actinobacteria*, and *Proteobacteria*. Clostridial cluster IV, *Lactobacillus, Caloramator, Clostridium, Sedimentibacter, Bacteroides* and *Porphyromonas* were active populations in situ, in which *Clostridial* cluster IV and *Clostridium* were likely involved in the hexanoate production.	Tao et al., 2017
Pit mud from 40 and 400 years old pits	Luzhou, Sichuan	Illumina MiSeq sequencing	*Methanobrevibacter, Caproiciproducens, Petrimonas, Lactobacillus, Sedimentibacter, Proteiniphilum, Syntrophomonas, Aminobacterium, Christensenellaceae* R-7, *Caldicoprobacter*, and *Olsenella* were the dominate genera. PM hosts a large number of novel taxa. The class *Clostridia* presented the highest proportion of novel OTUs.	Liu M.-K. et al., 2017
Zaopei fermented 0, 1, 4, 7, and 10 weeks	Sichuan	DGGE and gene clone	Diversity of bacteria in *Zaopei* decreased and after 1 week, only one bacterium phenotype was dominant. *Lactobacillus acetotolerans* appeared to play a key role during Chinese liquor fermentation.	Zhang et al., 2005
Zaopei fermented 0, 1, 4, 7 and 10 weeks, from the center and edge of the middle layer of the pit	Sichuan	DGGE and gene clone	*Issatchenkia, Talaromyces, Aspergillus* and *Eurotium* were the main dominant during the fermentation process. *Talaromyces*, and *Issatchenkia* were dominant fungal communities during the early stage of fermentation. After 4 weeks of fermentation, *Talaromyces, Eurotium*, and *Aspergillus*, became dominant.	Zhang et al., 2007
Multiple grains or single grains zaopei collected from the top layer and bottom layer of pits	–	DGGE and culture method	*Debaryomyces, Pichia* and *Candida* were dominant in multiple-grains zaopei. *Candida* was dominant in single-grains zaopei. *Thermophilic fungi* (*Thermomyces lanuginosus* and *Thermoascus aurantiacus*) were detected. Fungi communities in the top layer were richer than those in the bottom	Shi et al., 2011
Zaopei fermented 5, 20, and 40 days in summer or winter	Sichuan Province	Illumina Miseq sequencing	Bacterial population was mainly represented by *Acetobacter* and *Lactobacillus* both in winter and summer zaopei. The summer zaopei contained significantly higher proportions of LAB and lower proportions of *Acetobacter* than winter zaopei. *Thermoactinomycetaceae, Prevotella, Alcaligenes*, and *Gluconacetobacter* were identified	Sun et al., 2016
Pit mud, zaopei, and huangshui from new, 5-year, and 20-year pits	Yibin, Sichuan	FISH, PLFA, PCR-DGGE	*Lactobacillus, Clostridium, Sedimentibacter, Eubacterium*, uncultured bacterium were dominated in pit mud. *Lactobacillus, Clostridium, Sedimentibacter*, and uncultured bacterium were dominated in zaopei. *Methanobrevibacter, Methanocorpusculum, Methanoculleus, Saccharomycopsis*, and *Galactomyces* were detected in all samples.	Li et al., 2017
Mature daqu, pit mud, and zaopei fermented for 3, 15, and 45 days	Hunan	Illumina sequencing	*Lactobacillus, Leuconostoc, Staphylococcus, Gluconobacter, Acetobacter, Petrimonas, Clostridium, Ruminococcus, Methanobacterium* and *Methanobrevibacter* were dominant in 3 days’ zaopei. *Lactobacillus* was the predominant genus in 15 and 45 days’ zaopei. *Methanobacterium, Methanobrevibacter, Methanoculleus, Methanosarcina, Petrimonas, Lactobacillus, Sedimentibacter, Clostridium, Ruminococcus, Syntrophomonas*, and *Symbiobacterium* were dominant in pit mud. *Micromonospora, Petrimonas, Staphylococcus, Thermoactinomyces, Pediococcus, Lactobacillus, Leuconostoc, Weissella, Lactococcus, Sedimentibacter, Clostridium, Ruminococcus, Pantoea*, and *Pseudomonas* were dominant in daqu.	Wang X. et al., 2017
Huangshui from 20-year old pit	Sichuan	SSU rRNA library	*Proteobacteria, Firmicutes, Bacteroidetes, Lentisphaerae, Actinobacteria, Tenericutes*, and an unclassfied domain, respectively. The *Firmicutes* and *Proteobacteria* were the dominant in yellow water. The *Clostridium, Lactobacillus*, and *Serratia* were the dominant genus. Archea community in yellow water mostly consisted of genera *Methanosarcina* and *Methanoculleus*.	Li K. et al., 2015

*^#^, not available.*

### Microbial Diversity of Daqu

Daqu is the saccharification and fermentation agent used in the process of SFB production (Zheng et al., 2011). Daqu provides: microbial strains responsible for carrying out SFB fermentation. It also provides hydrolytic enzymes that can hydrolyze the macromolecules present in the fermented cereals; and flavoring compounds that act as precursors of SFB; as well as part of the fermentation material (Hu et al., 2004).

As far as bacterial diversity of daqu is concerned, *Bacillus* species have been detected in various SFB distilleries, which were analyzed with both culture-dependent as well as culture-independent methods. Among the various species identified, *B. licheniformis* was found to be the most common (Bin et al., 2011; Lin et al., 2012; Li et al., 2014; Zhang et al., 2014; Zheng X.W. et al., 2015). *B. licheniformis* present in daqu were found to secrete amylase (Li et al., 2014), protease, and some flavoring compound precursors (Yan et al., 2015). A recent study showed that inoculation of *B. licheniformis* in daqu fermentation changed the entire microbial community structure and metabolic profile of daqu (Wang P. et al., 2017).

In addition to *B. licheniformis*, daqu was also found to contain LAB (predominantly *Lactobacillus*) (Bin et al., 2011; Wang H.Y. et al., 2011). Members of LAB are known to be the main producers of lactic acid, which subsequently helps in the synthesis of ethyl lactate (via esterification). Apart from these, other genera detected in daqu included *Pseudomonas, Pantoea, Enterobacter, Klebsiella, Leuconostoc, Erwinia, Geobacillus, Weissella*, and *Staphylococcus* (Wang H.Y. et al., 2011; Gou et al., 2015; Zheng X.W. et al., 2015). Furthermore, actinomycetes, mostly *Thermoactinomyces* were detected as the dominant bacteria in daqu (Gou et al., 2015; Huang et al., 2016).

Understandably, yeasts are necessary for the production of alcohol. The yeast species present in SFB daqu can be divided into two groups: (i) those responsible for the production of ethanol (*Saccharomyces* that convert glucose into ethanol); and (ii) those responsible for the production of different kinds of esters (flavoring compounds of SFB), e.g., *Pichia*. The most dominant yeast varieties identified from SFB daqu included *Saccharomycopsis* (Wang H.Y. et al., 2011; Zhang et al., 2014; Huang et al., 2016), *Pichia* (Wang H.Y. et al., 2011; Zhang et al., 2014), and *Wickerhamomyces* (Zhang et al., 2014). *Saccharomycopsis fibuligera* was the most dominant yeast in SFB daqu (Wang H.Y. et al., 2011; Zhang et al., 2014; Huang et al., 2016) and was found to secrete amylases, acid proteases, and β-glucosidases, which in turn helped in starch degradation and their subsequent alcoholic fermentation (Chi et al., 2009). *Wickerhamomyces anomalus* was found to produce intra- and extracellular glucoside hydrolases, arabinosidase, and xylosidase (Sabel et al., 2014). These enzymes are highly important for the wine aroma of SFB. Other non-*Saccharomyces* yeast species found in SFB daqu included *Hanseniaspora, Issatchenkia, Trichosporon, Debaryomyces*, and *Sporidiobolus* (Wang H.Y. et al., 2011; Luo et al., 2013). In addition to yeast, molds were also found to dominate SFB daqu. *Aspergillus* [from the surface of wheat (Xu et al., 2004) or brewing workshop (Wang et al., 2012)] and *Lichtheimia* [isolated via culture-based method (Yang J.-G. et al., 2017)], were detected to be the most commonly found species in different SFB daqu samples (Wang H.Y. et al., 2011; Luo et al., 2013; Gou et al., 2015; Zhang X. et al., 2015). Furthermore, *Thermomyces, Thermoascus, Absidia*, and *Geotrichum* were identified as the most commonly occurring mold species that could not be isolated via the culture-based methods.

### Microbial Diversity of Pit Mud

SFB is usually produced by fermentation of cereals in an underground mud pit. The inside of these pit walls are covered with pit mud (PM), which significantly contributes toward maintaining the microbial diversity necessary for SFB fermentation. The microbial community of PM is most dominantly composed of eubacteria (especially, gram-positive and anaerobic bacteria; Zhao et al., 2012), archaea and fungi. The results obtained from these culture-independent methods indicated the dominant presence of *Firmicutes, Proteobacteria, Bacteroidetes, Actinobacteria*, and *Synergistetes* from the eubacteria domain and some Unclassified Bacteria (Ding et al., 2014b; Luo et al., 2014b; Liang et al., 2015). It is noteworthy that *Firmicutes* was found to predominate in many PM microbial communities (Guo et al., 2014; Luo et al., 2014a,b; Liang et al., 2015, 2016). Among *Firmicutes, Clostridiales, Lactobacillales*, and *Bacillales* were the main bacteria found in different SFB PMs (Zheng et al., 2013; Liang et al., 2015; Zhang L. et al., 2015). Furthermore, *Clostridium* (order Clostridiales) was detected as one of the most predominant bacteria in the PM microbial community and many specifies belonging to the genus *Clostridium* have already been isolated and identified from PM (Ding et al., 2014a; Tao et al., 2014; Zheng et al., 2015; Hu et al., 2016; Liang et al., 2016; Li et al., 2017). Some of the most common examples of such species include *Clostridium kluyveri* (Hu et al., 2015), *C. swellfunianum* (Liu et al., 2014), *C. butyricum* (Li et al., 2016), and *C. liquoris* (Yin et al., 2016). It was also proposed that these members contribute in the: (1) production of organic acids (acetic, butyric, and hexanoic (caproic) acid), which then gets esterified with ethyl alcohol via enzymatic and non-enzymatic catalysis to form ethyl butyrate and caproate; (2) production of H_2_ for the synergistic metabolism of methanogens. Meanwhile, *Ruminococcus, Syntrophomonas, Desulfotomaculum*, Anaerobrancaceae, *Pelotomaculum, Eubacterium*, and *Butyrivibrio* were identified as the most dominant members of *Clostridiales* (Ding et al., 2014a; Hu et al., 2014, 2016; Tao et al., 2014; Zheng et al., 2015; Liang et al., 2016; Li et al., 2017). On the other hand, *Lactobacillus*, especially *Lactobacillus acetolerans, Lb. alimentarius* and *Lb. acetolerans* (Zheng et al., 2013), followed by *Lactococcus* (Liang et al., 2016), were the most dominant of all *Lactobacillales* found in PMs (Ding et al., 2014a; Tao et al., 2014; Zheng et al., 2015; Hu et al., 2016; Liang et al., 2016; Li et al., 2017). Interestingly, *Bacillales*, especially those belonging to the genus *Bacillus*, were mostly detected in young PMs (Ding et al., 2014b; Liang et al., 2016). *Virgibacillus* is also detected (Ding et al., 2014b). Other commonly detected bacterial strains belonged to the phylum *Firmicutes*, and included members of *Sedimentibacter* (Ding et al., 2014b; Tao et al., 2014; Zheng et al., 2015; Hu et al., 2016; Li et al., 2017), *Sporanaerobacter* (Hu et al., 2016), and *Tissierella* (Liang et al., 2016).

Apart from *Firmicutes*, other commonly observed bacterial species found in PM included: *Proteobacteria, Pseudomonas* (Liang et al., 2016), *Bacteroidetes, Petrimonas, Prevotella* (Tao et al., 2014; Hu et al., 2016), *Chloroflexi* (Luo et al., 2014a), *Actinobacteria, Rhodococcus, Microbacterium, Acinetobacter* (Ding et al., 2014b; Liang et al., 2016), *Synergistetes, Altererythrobacter* (Liang et al., 2016), and *Aminobacterium* (Zheng et al., 2015; Liang et al., 2016). It is noteworthy that *Chloroflexi, Synergistetes*, and *Actinobacteria* were only detected in aged PMs (Luo et al., 2014a,b).

Among the various categories of fungal strains, those belonging to the order Saccharomycetales were found to be the most dominant (Zhang L. et al., 2015). *Pichia, Wickerhamomyces, Saccharomyces*, and *Galactomyces* were found to constitute a major portion of the core fungal strains isolated from different PM samples (Zheng et al., 2013, 2015; Ding et al., 2015; Li et al., 2017; Liu M. et al., 2017). In addition, *Kluyveromyces* (Zheng et al., 2013), *Zygosaccharomyces, Geotrichum* (Ding et al., 2015), *Saccharomycopsis, Issatchenkia* (Li et al., 2017), and *Debaryomyces* (Liu M. et al., 2017) were also detected. *Aspergillus* is another fungal genus that was categorized as the chief constituent of the fungal diversity of PMs (Ding et al., 2015; Zheng et al., 2015; Liu M. et al., 2017). Other genera that formed an integral part of core fungal diversity included *Rhizopus, Phoma, Trichosporon, Thermoascus, Penicillium, Thermomyces, Malassezia, Mucor, Davidiella, Wallemia, Toxicocladosporium, Fusarium*, and *Cladosporium* (Liu M. et al., 2017). Among them, *Rhizopus, Phoma*, and *Trichosporon* were found to be relatively richer in young PM samples, while *Aspergillus* and *Candida* were in older PM samples (Liu M. et al., 2017).

*Methanobrevibacter, Methanobacterium*, and *Methanoculleus* were found to be the most predominant genera of the domain archaea that were found in different PMs (Ding et al., 2014a,b, 2015; Luo et al., 2014b; Tao et al., 2014; Wu et al., 2015; Zheng et al., 2015; Hu et al., 2016; Li et al., 2017). Few other studies indicated the presence of members of *Methanosaeta, Methanosarcina* (Ding et al., 2014b, 2015; Luo et al., 2014b; Tao et al., 2014; Wu et al., 2015; Zheng et al., 2015; Hu et al., 2016)., *Nitrososphaera* (Hu et al., 2016), *Methanocorpusculum* (Ding et al., 2015; Li et al., 2017), *Methanocorpusculum* (Wu et al., 2015), *Methanoplanus, Methanotorris, Methanolobus, Methanothermobacter*, and *Methanomethylovorans* (Zheng et al., 2015).

### Microbial Diversity of Zaopei

Zaopei represents the fermented cereals that are placed inside the pit cellar for alcohol fermentation and formation of flavoring compounds. Typically, fresh zaopei is essentially a mixture of steamed cereals, steamed rice husks, and daqu powder (**Figure 1**). Microorganisms enter into the zaopei via daqu powder, PM and the ambient brewing workshop environment. The primary role of the bacteria existing in zaopei is to produce varieties of flavoring compounds or precursor of those compounds, such as caproic acid, lactic acid, and butyric acid. It was found that these bacteria mostly belonged to the *Lactobacillaceae* and *Acetobacteraceae* families (Zhang et al., 2005; Sun et al., 2016; Li et al., 2017). Subsequent studies indicated that *Lactobacillus acetotolerans* was the most dominant strain present in zaopei (Zhang et al., 2005). Other members of LAB that were detected were *Streptococcus, Lactococcus, Leuconostoc*, and *Weissella* (Zhang et al., 2005; Sun et al., 2016). Apart from *Lactobacillus, Acetobacter* was found to be another chief microbial constituent of zaopei (Sun et al., 2016). In addition, members of *Bacillus* were found to be the main bacterial strains that were isolated and cultured from zaopei (Wang T. et al., 2011). *Clostridium*, which is a dominant bacterium in PM, was also identified in zaopei (Zhang et al., 2005; Sun et al., 2016; Li et al., 2017). Other not so dominant genera identified in zaopei were *Erwinia, Kozakia, Staphylococcus, Granulicatella, Arthrobacter, Microbacterium, Shewanella, Sporolactobacillus, Thermoactinomyces, Desmospora, Alcaligenes, Gluconacetobacter, Prevotella*, and *Sedimentibacter* (Zhang et al., 2005; Sun et al., 2016; Li et al., 2017).

As far as fungal diversity is concerned, members of the order *Saccharomycetales* were identified as the main fungal strains present in different zaopei samples (Shi et al., 2011; Li et al., 2017). *Candida, Issatchenkia, Debaryomyces*, and *Pichia* were reported as the main fungal genera identified in it. Other *Saccharomycetales* found in the zaopei included: *Torulaspora, Zygosaccharomyces, Saccharomycopsis, Citeromyces, Galactomyces, Hyphopichia, Cyberlindnera, Geotrichum, Magnusiomyces*, and *Kluyveromyces* (Zhang et al., 2007; Shi et al., 2011; Li et al., 2017). Some other dominantly present fungal species reported are: *Talaromyces, Aspergillus, Eurotium* (Zhang et al., 2007), *Fomitopsis, Trichosporon, Thermomyces*, and *Thermoascus* (Zhang et al., 2007; Shi et al., 2011).

In addition, five genera from archaea, namely *Methanocorpusculum, Methanobrevibacter, Methanobacterium, Methanoculleus* (most dominant; Ding et al., 2015), and *Methanosaeta* were also detected in zaopei (Sun et al., 2016; Li et al., 2017).

### Microbial Diversity of Huangshui

Huangshui (HS) is the brown viscous liquid which is formed by the liquid that permeates to the bottom of pit during the fermentation process. It was found to be full of microbial strains that have evolved through long term domestication. *Lactobacillus* and *Clostridium* were identified as the dominant bacterial genera present in HS (Ding et al., 2015; Li K. et al., 2015; Li et al., 2017). Other commonly occurring genera detected in HS included *Acetobacter, Proteiniphilum*, and *Caloramator* (Ding et al., 2015; Li K. et al., 2015; Li et al., 2017). *Methanocorpusculum, Methanoculleus, Methanosarcina, Methanobrevibacter, Methanobacterium*, and *Methanosaeta* were the prevalently detected genera belonging to archaea in HS, out of which the former two genera were the most dominant (Ding et al., 2015; Li K. et al., 2015; Li et al., 2017). In addition, fungal genera viz. *Aspergillus, Geotrichum, Galactomyces, Pichia, Zygosaccharomyces*, and *Candida* were also detected in HS (Ding et al., 2015; Li et al., 2017), among which *Pichia* was found to be the most dominant (Ding et al., 2015).

## Microbial Community Dynamics in SFB Ecosystem

For the microbial community dynamics of daqu, the rapid propagation of most bacterial taxa (especially *Lactobacillales*), and several fungal taxa containing *Candida, Wickerhamomyces*, unclassified *Dipodascaceae*, and *Saccharomycetales* significantly enhanced the temperature at the initial fermentation stage (Xiao et al., 2017). *Wickerhamomyces anomalus, Candida metapsilosis* were isolated and identified as the main yeasts, and *Rhizopus oryzae* as the main molds in this stage (Yang J.-G. et al., 2017). When the fermented temperature increased to highest values (about 55°C), thermotolerant taxa including *Bacillus*, unclassified *Streptophyta, Weissella, Thermoactinomyces, Thermoascus*, and *Thermomyces, Saccharomycetales*, and no-rank *Eukaryota* were dominant (Huang et al., 2017b; Xiao et al., 2017). For mature daqu, *Bacillales* and *Eurotiales* became the dominant bacterial and fungal taxon, respectively (Huang et al., 2017b). After 10 days fermentation, *Saccharomycopsi fibuligera, Bacillus subtilis* subsp. *inaquosorum, Lichtheimia ramose* were isolated and identified as the dominant yeast, bacterium, and mold, respectively (Yang J.-G. et al. 2017). In addition, Lactic acid bacteria related to *Weissella, Leuconostoc*, and *Lactobacillus* were dominant bacteria through fermentation (Xiao et al., 2017). *Thermoascus, Candida, Wickerhamomyces*, and *Thermomyces* were dominant fungal genera through fermentation (Xiao et al., 2017).

For the microbial community dynamics in zaopei, *Lactobacillus, Leuconostoc, Methanobacterium, Clostridium, Acetobacter, Gluconobacter, Staphylococcus, Petrimonas, Methanobrevibacter*, and *Ruminococcus* were the main prokaryotic genera at the beginning of fermentation (Wang X. et al., 2017). After 2 weeks fermentation, *Lactobacillus* became dominant, and other bacterial genera decreased (Wang X. et al., 2017). At the end of zaopei fermentation, only *Lactobacillus* was absolutely dominant (Zhang et al., 2005; Wang X. et al., 2017). However, culture method identified that *Bacillus* and *Lactobacillus* were both the dominant bacterial genera during the zaopei fermentation (Dou et al., 2017). *Talaromyces* and *Issatchenkia* are the dominant fungus at the beginning stage, *Eurotium* and *Aspergillus* became the dominant genera after 4 weeks fermentation (Zhang et al., 2007). However, *Saccharomyces cerevisiae*, the main ethanol producer, was not identified as the dominant species during the fermentation by the sequencing method, which was not in accordance with the result obtained by traditional culture methods (Yang J. et al., 2017).

## Systems Biology Based Analysis of SFB Microbial Community

The diversity of microbial community of the SFB ecosystem has been widely studied in the past few years (**Tables 1, 2**). However, a comprehensive understanding of such complex microbial communities necessitates establishing links between the active microbial diversity and their functional aspects (Maukonen and Saarela, 2009). Until now, the metabolic function of microbial community of SFB ecosystem was mainly studied by analyzing the physiological and biochemical features of the isolated microorganisms. This method is not only labor intensive and time consuming, the results obtained are also limited to the number of culturable microbes. Fortunately, the advent of highly efficient contemporary sequencing technology, omics based technologies as well as corresponding bioinformatics software and database have greatly promoted the study on the functions of even the uncultured microbial communities present in traditional food ecosystems, such as SFB ecosystem (Franzosa et al., 2015; Chen et al., 2017).

Several omics based research studies were carried out to elucidate the microbial community in SFB ecosystem. On the species level, genomes of *Rhizopus chinensis* from daqu, *C. kluyveri* and *C. butyricum* from PM have already been sequenced (Wang D. et al., 2013; Li et al., 2016). It is proposed that the genomic sequence analysis of these three microorganisms can help in achieving better understanding of their genetic background and potential functions. On the microbial community level, Tao et al. (2017) applied the metagenomic approach and MiSeq-sequencing analyses of 16S rDNA and 16S rRNA genes to identify the hexanoate producing microorganisms (*Clostridial* cluster IV and *Clostridium*) in the SFB ecosystem. They also achieved the detailed elucidation of the interspecies hydrogen transfer mechanisms between hexanoate-producing bacteria and methanogens in PM microbiome. Zheng et al. (2015) investigated the aroma-forming functional proteins in PM samples by using the iTRAQ-based proteomic technology. The results thus obtained indicated that most proteins were involved in the process of methanogenesis, and caproic and butyric acid formation. Furthermore, it was found that these results were in accordance with the data obtained from metagenomic analysis (Tao et al., 2017).

Huang et al. (2017a) studied the metabolism and functional enzymes of the active microbial communities in SFB daqu via the metatranscriptomics approach. It was found that key enzymes involved in glycolysis and starch as well as pyruvate and ethanol metabolism were over-expressed at 50 and 62°C. Furthermore, the citrate cycle was up-regulated at 62°C and all the up-regulated genes in the glycolysis pathways mainly belonged to Saccharomycetales and Mucorales (Huang et al., 2017a). Liu J. et al. (2017) implemented the metatranscriptomic analysis approach to identify that *Saccharomyces* and *Lactobacillus* were the core microbiota responsible in the sulfur compound production mechanism in zaopei. These studies helped in providing a detailed description of the biological components of an active SFB ecosystem. It was then proposed that correlating these components with the functional aspects of the microbial community needs to be accomplished, which can be achieved by integrating these multi-omics data with systems biology approaches (Franzosa et al., 2015). Data so obtained (on various functional levels) can be implemented for the construction and analysis of a community-level metabolic model with predictable capability (Biggs et al., 2015; Cardona et al., 2016).

## Interactions in Microbial Community of SFB Ecosystem

It was found that there existed extensive interspecies interactions among microbial communities of SFB ecosystem (Li et al., 2017). Cooccurrence pattern analysis suggested the presence of potential synergetic relationships between members belonging to the genera: *Clostridia, Bacteroidia, Methanobacteria*, and *Methanomicrobia* (Hu et al., 2016). It was also suggested that these relationships may be beneficial for the stability of the PM ecosystem. However, LAB (*Lactobacillus, Pediococcus*, and *Streptococcus*) may destroy this stability by producing lactate or various bacteriocins (Hu et al., 2016). Moreover, in zaopei ecosystem, *Lactobacillus* was found to be negatively related with the occurrence of *Clostridium, Ruminococcus, Sedimentibacter, Syntrophomonas, Thermoactinomyces, Leuconostoc, Pediococcus, Staphylococcus, Bacillus*, and *Lactococcus* (Wang X. et al., 2017). Synergistic interactions between the hexanoate producing strains (mostly, genus *Clostridium*) and methanogenic archaea (*Methanobacterium*) were also detected (Barker and Taha, 1942; Wu et al., 1990; Tao et al., 2017) and the main mechanism was the interspecies hydrogen transfer (Thauer et al., 2008). The caproic acid biosynthesis and hydrogenotrophic and acetoclastic methanogenesis pathways were also detected in a recent metagenomics analysis of PM microbial community (Tao et al., 2017). In addition, co-culture of *Clostridium* sp. W1 and *Methanobacterium bryantii* had been used for the cultivation of PM in new pit cellar, which resulted in a higher caproate and ethylcaproate production capacity (Wu et al., 1990). Another example is that the production of caproic acid in *Clostridium* was enhanced by the melanin secreted by *Streptomyces avicenniae* GW01 (Guo et al., 2016).

Recently, a novel synergistic effect between *Saccharomyces* and *Lactobacillus* in the production of sulfur compounds via methionine recycling was identified (Liu J. et al., 2017). It was found that the presence of *L. buchneri* up-regulated the expression of genes responsible for the generation of 3-(Methylthio)-1-propanol and dimethyl disulfide in *S. cerevisiae*, which further regenerated the precursor of methionine catabolism (Liu J. et al., 2017). Though, several examples of interspecies interactions have been identified, but the detailed mechanisms of the same are still unclear.

It has also been observed that some *Aminobacterium* strains and *Methanosarcina barkeri* can enhance the generation of VFAs, which further contributes to the aroma composition of Chinese liquor (Nadell et al., 2016). Furthermore, *Bacillus* species were found to inhibit the growth of *Streptomyces sampsonii*, which is one of the most dominant geosmin producers (Zhi et al., 2016).

## Flavor Contributions of Microbial Community in SFB Ecosystem

To full exploration of SFB ecosystem, one key is to understand function of microbial community in SFB ecosystem attributing to flavor compounds formation. Because flavor compounds of SFB are complex, the first thing is to identify the characteristic flavor compounds of SFB. Ethyl esters (ethyl hexanoate, ethyl lactate, ethyl acetate, and ethyl butanoate), acids (lactic acid, acetic acid), alcohols (*n*-propanol, iso-butanol), aldehyde had been reported as the main flavor compounds in SFB (Zhou et al., 2012; Li J. et al., 2015; Yao et al., 2015). At present, studies on the flavor contributions of microbial community in SFB ecosystem can be divided into three groups: (i) isolation and identification of microorganism with high flavor compound producing capacity under pure liquid culture, (ii) regulation of SFB ecosystem with addition of microorganisms during fermentation, and (iii) detection and analysis of biological components of SFB ecosystem on a certain level with the systems biology approaches. The first and third groups have been discussed in the above sections: “Physiological traits of cultured microorganisms” and “Systems biology based analysis of SFB microbial community.” For the second group, addition of yeast strains (*Debaryomyces hansenii, Issatchenkia orientalis, Zygosaccharomyces bailii, Trichosporon coremiiforme*) to zaopei showed an increase of ethyl hexanoate (Wang T. et al., 2013). Inoculation of *Wickerhamomyces anomalus* showed an increase of amino nitrogen, n-butyl alcohol, and sec-butyl alcohol in zaopei (Jian et al., 2017). However, inoculation of *Saccharomyces cerevisiae* showed a decrease of esters compounds and bacterial and fungal diversity in zaopei (Wang S. et al., 2017). For further study, flavor–oriented technology, which integrates the identification of flavor compounds profile and characteristic flavor compounds, critical microbes and their metabolic features, regulation of flavor production of ecosystem by the critical microbes, has been carried out and showed great potential (Xu, 2015).

## Conclusion and Perspectives

SFB are produced by the synergistic effect of various microorganisms present in the SFB ecosystem. Due to the rich microbial diversity of the SFB ecosystem, it can be considered as a potential resource for the isolation of many potential industrial microorganisms that are capable of producing organic acids, novel enzymes and other high-value products. In addition, the SFB ecosystem could be a good example for the study of microbial community formation (Wolfe and Dutton, 2015) and microbial adapt evolution. Up until now, most of the microbial diversity studies on the SFB ecosystem were focused on the isolation and culture of the functional microbes and identification of microbial diversity of SFB ecosystem. However, the results obtained from such studies fail to provide comprehensive overview of the function and interspecies interactions of the microbial community of the SFB ecosystem. It was then observed that such culture based methods fail to study many un-cultured microorganisms, due to which their potential roles remain ambiguous (Liu M.-K. et al., 2017). Furthermore, the isolated microorganisms may behave differently under axenic liquid and solid culture conditions and fail to provide crucial information on the interspecies interactions associated with them (Zhou et al., 2016). New cultivation methods should be developed to identify phenotypes of specific species and elucidate physiological interactions of these microbes and their functions in SFB ecosystem (Sommer, 2015; Liu J. et al., 2017).

The contemporary application of systems biology approaches has boosted our understanding of SFB ecosystem, not only the microbial diversity, but also the potential metabolic functions (Huang et al., 2017a; Tao et al., 2017). It is therefore proposed that a detailed explanation of microbial community of SFB ecosystem calls for a combination of prevalent omics data, results obtained from physiological experiments, application of molecular analysis methods, systems biology approaches as well as bioinformatics tools. In the near future, we believe that the persistent investigation on the molecular mechanisms of microbial constituents of the SFB ecosystem will help in accelerating the improvement of SFB quality and stability.

## Author Contributions

WZ carried out the initial literature review and wrote the initial manuscript. HL provided expertise and insight relating to baijiu microbiology. WZ and CZ revised the text. All authors read and approved the final manuscript.

## Conflict of Interest Statement

The authors declare that the research was conducted in the absence of any commercial or financial relationships that could be construed as a potential conflict of interest.
